# Identification of protein–protein interaction associated functions based on gene ontology and KEGG pathway

**DOI:** 10.3389/fgene.2022.1011659

**Published:** 2022-09-12

**Authors:** Lili Yang, Yu-Hang Zhang, FeiMing Huang, ZhanDong Li, Tao Huang, Yu-Dong Cai

**Affiliations:** ^1^ Measurement Biotechnique Research Center, School of Biological and Food Engineering, Jilin Engineering Normal University, Changchun, China; ^2^ Channing Division of Network Medicine, Brigham and Women’s Hospital, Harvard Medical School, Boston, MA, United States; ^3^ School of Life Sciences, Shanghai University, Shanghai, China; ^4^ Bio-Med Big Data Center, CAS Key Laboratory of Computational Biology, Shanghai Institute of Nutrition and Health, University of Chinese Academy of Sciences, Chinese Academy of Sciences, Shanghai, China; ^5^ CAS Key Laboratory of Tissue Microenvironment and Tumor, Shanghai Institute of Nutrition and Health, University of Chinese Academy of Sciences, Chinese Academy of Sciences, Shanghai, China

**Keywords:** protein-protein interaction, gene ontology, KEGG pathway, enrichment, feature analysis

## Abstract

Protein–protein interactions (PPIs) are extremely important for gaining mechanistic insights into the functional organization of the proteome. The resolution of PPI functions can help in the identification of novel diagnostic and therapeutic targets with medical utility, thus facilitating the development of new medications. However, the traditional methods for resolving PPI functions are mainly experimental methods, such as co-immunoprecipitation, pull-down assays, cross-linking, label transfer, and far-Western blot analysis, that are not only expensive but also time-consuming. In this study, we constructed an integrated feature selection scheme for the large-scale selection of the relevant functions of PPIs by using the Gene Ontology and Kyoto Encyclopedia of Genes and Genomes (KEGG) pathway annotations of PPI participants. First, we encoded the proteins in each PPI with their gene ontologies and KEGG pathways. Then, the encoded protein features were refined as features of both positive and negative PPIs. Subsequently, Boruta was used for the initial filtering of features to obtain 5684 features. Three feature ranking algorithms, namely, least absolute shrinkage and selection operator, light gradient boosting machine, and max-relevance and min-redundancy, were applied to evaluate feature importance. Finally, the top-ranked features derived from multiple datasets were comprehensively evaluated, and the intersection of results mined by three feature ranking algorithms was taken to identify the features with high correlation with PPIs. Some functional terms were identified in our study, including cytokine–cytokine receptor interaction (hsa04060), intrinsic component of membrane (GO:0031224), and protein-binding biological process (GO:0005515). Our newly proposed integrated computational approach offers a novel perspective of the large-scale mining of biological functions linked to PPI.

## 1 Introduction

In living creatures, protein–protein interactions (PPIs) are one of the basic formats of molecular interactions that regulate various important biological functions, including cell proliferation, differentiation, and apoptosis. Traditionally, PPIs can be identified by using experimental methods, such as co-immunoprecipitation, pull-down assays, cross-linking, label transfer, and far-Western blot analysis (Hall, 2015; Evans and Paliashvili, 2022; Lyu et al., 2022). Various significant PPIs have been identified by using complex but accurate experiment-based methods. The identified PPIs can be divided into two groups: 1) PPIs that transport cell signals for downstream biological functions. For example, 14-3-3 protein complexes have been reported to interact as cell-signaling transporters with multiple protein molecules via PPIs to regulate inflammatory effects (Munier et al., 2021). 2) PPIs that establish stable complexes. The stable complex of ferrtin is formed by two subunits: the ferrtin heavy chain and the ferrtin light chain (Blankenhaus et al., 2019). Interactions between these two subunits form the stable ferrtin complex and further play a specific role in iron metabolism (Neves et al., 2019).

Although experiment-based approaches have been widely used to recognize various functional PPIs, they are not only expensive but also time-consuming. With the establishment of the PPI databases, advanced computational algorithms, especially machine learning methods, have been introduced to explore new PPIs and identify connections between biological functions and PPIs (Balogh et al., 2022; Gao et al., 2022; Ieremie et al., 2022). Three major aspects of PPIs have been widely reported with the application of machine learning methods: 1) Microbe–host protein interactions. Early in 2019, researchers summarized the optimized methods for selecting features to describe viral protein–host protein interactions; this effort indicated that microbe–host interactions can be predicted by using computational methods (Zheng et al., 2019). 2) Protein interactions in human malignant diseases, such as cancer. In 2020, predicted PPIs were applied to recognize glioma stages; this approach indicated that predicted PPIs can also predict disease progression and thus extended the application of PPIs based on machine learning models (Niu et al., 2020). 3) Predicted protein interactions in drug development. Through the integration of PPIs predicted by a machine learning method and drug physical scoring (Guedes et al., 2021), newly identified PPIs were shown to be robust for drug discovery and pharmalogical mechanism exploration.

Therefore, machine learning methods become more and more popular for new PPI recognition and PPI function exploration. They have been deemed to be one of the major novel tools for PPI studies. As introduced above, PPIs are one of the basic approaches for molecular interactions regulating essential biological functions in all living creatures. Machine learning methods can help recognize key functional potentials that can be attributed to PPIs. In this study, multiple machine learning methods were employed to conduct the investigation. First, each PPI was represented by lots of features derived from gene ontology (GO) terms or Kyoto Encyclopedia of Genes and Genomes (KEGG) pathways of two proteins in the PPI. Then, several machine learning methods, including Boruta (Kursa and Rudnicki, 2010), least absolute shrinkage and selection operator (LASSO) regression (Tibshirani, 1996), light gradient boosting machine (LightGBM) (Ke et al., 2017), and max-relevance and min-redundancy (mRMR) (Peng et al., 2005), were adopted to deeply analyze these features. Key features yielded by different methods were integrated by a comprehensive evaluation method to obtain most essential features. Their corresponding GO terms and KEGG pathways, such as cytokine–cytokine receptor interaction (hsa04060), intrinsic component of membrane (GO:0031224), and protein-binding biological process (GO:0005515), were analyzed to uncover their relationships to PPIs. This study reflected the important and irreplaceable roles of GO terms and KEGG pathways for PPIs.

## 2 Materials and methods

### 2.1 Data acquisition

All human PPIs used in this research were retrieved from STRING (https://string-db.org/, version 9.1) (Franceschini et al., 2013). These interactions were obtained through the following sources: high-throughput experiments, genomic context, (conserved) co-expression, and previous knowledge. PPIs with “Experimental” scores greater than zero were selected, which indicated that these PPIs had been experimentally confirmed. 309,287 human PPIs involving 16,571 proteins were accessed. However, if all of this PPI information was adopted, the subsequent calculations would introduce significant noise due to redundant protein sequences and unmanifested protein functions. The following screening processes were performed to create a well-defined PPI dataset: 1) By applying CD-HIT (Fu et al., 2012), similar proteins were excluded. The similarity of any two remaining proteins was less than 0.25. 2) Proteins without GO terms or KEGG pathways were also discarded. After the above filtering process, 6623 proteins and 70,392 pairs of PPIs were retained. These PPI comprised the positive sample set.

Pairs of proteins without PPIs are also necessary to study the specific function of PPIs. We randomly selected two proteins from the 6623 proteins obtained through the above screening to constitute pairs of PPIs. If the pair did not exist in the positive sample set, it was treated as a negative sample. Through random combination, 21,928,753 pairs can be obtained, including 21,858,361 negative samples and 70,392 positive samples. However, the considerably higher number of negative samples than that of positive samples indicated that the constructed dataset was extremely imbalanced. Direct analysis of such imbalanced dataset would produce bias. As the negative samples were 310 times as many as positive samples, the negative samples were divided into 310 subsets randomly and equally. Each subset was combined with the positive sample set to form a balanced learning dataset. As a result, 310 datasets for subsequent analysis were created.

### 2.2 Representation of protein–protein function associations

GO terms and KEGG pathways are well-known functional information for deciphering and describing the molecular functions, cellular components, and biological processes of proteins or genes (Kanehisa et al., 2012; Gene Ontology Consortium, 2015). As in our prior study, we used such functional terms (GO terms and KEGG pathways) of proteins to generate the representations of PPIs (Yuan et al., 2019; Zhang et al., 2021). Based on the GO information of a protein p , it can be encoded as

$$v_{GO}\left(p\right)={\left[g_{1}^{p},g_{2}^{p},\ldots ,g_{n}^{p}\right]}^{T},$$ (1)

where n is the total number of GO terms (*n* = 17916 in this study). $g_{i}^{p}$ equals 1 if the protein p is annotated by the i -th GO term. Otherwise, $g_{i}^{p}$ equals 0. Likewise, p can be encoded as the following vector using its KEGG pathway information

$$v_{kegg}\left(p\right)={\left[k_{1}^{p},k_{2}^{p},\ldots ,k_{m}^{p}\right]}^{T},$$ (2)

where $g_{i}^{p}$ and $k_{i}^{p}$ are also similar in value, and *m* stands for the number of pathways (*m* = 279 in this study). For a PPI, we cannot simply combine the features of two proteins when generating the features of PPI because the order information of the PPI should be excluded. We utilized the following scheme, which has been employed in some studies (Chen et al., 2013; Ran et al., 2022), to construct the feature vectors of PPIs. The feature vectors for GO terms and KEGG pathways of a PPI consisting of $p_{1}$ and $p_{2}$ were constructed by using the following scheme:

$$V_{GO}\left(PPI\right)=v_{GO}\left(p_{1}\right)⊗v_{GO}\left(p_{2}\right)={\left[g_{1}^{p_{1}}+g_{1}^{p_{2}},\left|g_{1}^{p_{1}}-g_{1}^{p_{2}}\right|,\ldots ,g_{n}^{p_{1}}+g_{n}^{p_{2}},\left|g_{n}^{p_{1}}-g_{n}^{p_{2}}\right|\right]}^{T},$$ (3)

$$V_{KEGG}\left(PPI\right)=v_{KEGG}\left(p_{1}\right)⊗v_{KEGG}\left(p_{2}\right)={\left[k_{1}^{p_{1}}+k_{1}^{p_{2}},\left|k_{1}^{p_{1}}-k_{1}^{p_{2}}\right|,\ldots ,k_{m}^{p_{1}}+k_{m}^{p_{2}},\left|k_{m}^{p_{1}}-k_{m}^{p_{2}}\right|\right]}^{T}$$ (4)

By integrating above two feature vectors, we can finally represent the feature vector of the PPI as follows:

$$V\left(PPI\right)=V_{GO}\left(PPI\right)⊗V_{KEGG}\left(PPI\right)=\left[\begin{array}{c} V_{GO}\left(PPI\right)\\ V_{KEGG}\left(PPI\right) \end{array}\right]$$ (5)

### 2.3 Feature filtering with boruta

A large number of features were used to describe PPIs by using GO terms and KEGG pathways. Evidently, lots of features were unrelated to distinguish positive and negative samples, which must be filtered to reduce the noise in subsequent calculations. Here, Boruta was adopted to exclude irrelated features and retain relevant ones.

Boruta, a wrapper-based feature selection method, uses random forest as the classifier to filter out a set of features that are relevant to the target variable (Kursa and Rudnicki, 2010; Zhang et al., 2020; Chen et al., 2021; Ding et al., 2021; Zhou et al., 2022). It is implemented through the following steps: 1) The features are randomly shuffled and then stitch together with the actual feature matrix to form a new feature matrix. 2) The importance of the shuffled and actual features is obtained by inputting the new feature matrix into the random forest. 3) The actual features with importance greater than the maximum importance of the shuffled features are retained. By iterating the above steps several times, the important features are identified by Boruta.

For this study, the Boruta program retrieved from https://github.com/scikit-learn-contrib/boruta_py was used, which was executed with its default parameters on each of 310 datasets.

### 2.4 Feature ranking algorithms

Through Boruta, some relevant features can be screened out. However, their contributions for distinguishing positive and negative samples were not same. They should be further analyzed. Here, we ranked these features in accordance with their importance by using three efficient feature ranking algorithms: LASSO (Tibshirani, 1996), LightGBM (Ke et al., 2017), and mRMR (Peng et al., 2005). These feature ranking algorithms are briefly described as below.

In 1996, Tibshirani et al. proposed the LASSO algorithm, which is primarily used to select variables (Tibshirani, 1996). The LASSO method constructs a regression model by employing a penalty function with coefficients, each of which corresponds to one feature. The coefficients of features can be an indicator to measure the importance of features. Accordingly, features can be ranked based on their corresponding coefficients. In this study, the LASSO package collected in Scikit-learn (Pedregosa et al., 2011) was adopted and applied to all 310 datasets for generating feature lists. Such obtained lists were called LASSO feature lists in this study.

LightGBM is a gradient boosting decision tree algorithm that was proposed by Ke et al., in 2017 (Ke et al., 2017; Ding et al., 2022). This method consists of multiple decision trees, and the weights of each tree are considered in the classification. The importance of a feature is determined by the number of times it is used in the constructed decision trees. Accordingly, features can be sorted in a list with the decreasing order of such times. The present study used the LightGBM program downloaded from https://lightgbm.readthedocs.io/en/latest/, which was performed on 310 datasets. For convenience, the lists yielded by LightGBM were called LightGBM feature lists.

The mRMR algorithm is a heuristic feature selection method in which the original features are ranked in accordance with a well-defined scheme (Peng et al., 2005; Wang et al., 2018; Zhao et al., 2018; Chen et al., 2022). This scheme considers that the importance of features is determined by two aspects: relevance to target variable and redundancies to other features. The feature with high relevance to target variable and low redundancies to other features should be assigned a high rank in the final feature list. A loop procedure determines the rank of all features. In each round, the feature with greatest difference between its relevance to target variable and redundancies to already-selected features is selected and appended to the list. This study adopted the mRMR program obtained from http://home.penglab.com/proj/mRMR/. It was executed on each of 310 datasets. The generated lists were termed as mRMR feature lists.

### 2.5 Comprehensive evaluation of feature lists

Given that the negative samples were randomly chosen and divided into 310 datasets, the features that were selected by Boruta from 310 datasets were distinctive. Given a certain feature ranking algorithm described in Section 2.4, 310 feature lists can be generated, denoted by $F_{1},F_{2},\cdots F_{310}$ . Features occurring in these lists were collected. For one feature *f*, its rank in $F_{i}$ was denoted by $R_{i}\left(f\right)$ . In particular, if the list did not contain this feature. Its rank was denoted by 0. Furthermore, count the number of lists containing feature *f*, denoted by N(f) . The importance of feature *f* was measured by the following importance score

$$Importance\;score\;\left(f\right)=\frac{M\left(f\right)}{W\left(f\right)},$$ (6)

where M(f) was the mean ranks of *f*, calculated by M(f)=∑i=1310Ri(f)N(f) , and W(f) represented the weight of *f*, defined as N(f)310 . The numerator in Eq. 6 considered the evaluation results yielded by the feature ranking algorithm on different datasets, whereas the denominator further considered the evaluation results of Boruta on different datasets. Generally, a high weight, i.e., the feature was selected by Boruta on many datasets, suggested the feature was important. In this case, the penalty, the reciprocal of weight, on the mean rank was small. Thus, the smaller the importance score, the more important the feature. All features were ranked in terms of the increasing order of their importance scores. Under such operation, 310 feature lists were integrated into one feature list.

As three feature ranking algorithms were used, three integrated feature lists can be obtained. Top 100 features in each integrated list were picked up. The features that ranked high in all three feature lists were most relevant to PPIs, which were valuable for giving detailed analysis.

## 3 Results

**FIGURE 1:**
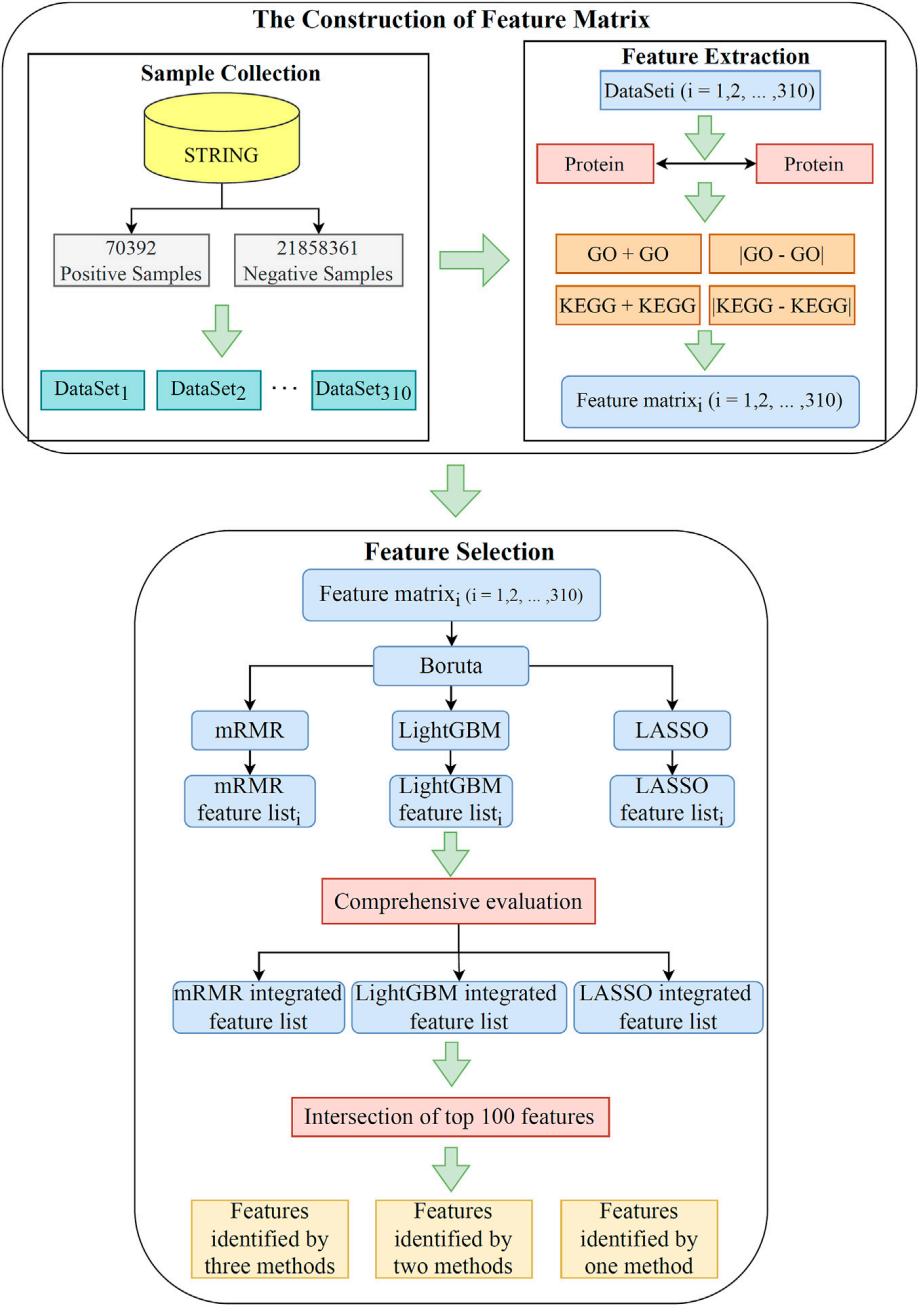
Flow chart of the whole analytical process. A total of 21,928,753 pairs of PPIs acquired from the STRING database are divided into 70,392 positive samples and 21,858,361 negative samples. The negative samples are randomly and equally divided into 310 subsets, yielding 310 datasets. Each dataset is characterized by using GO terms and KEGG pathways. Subsequently, the features in each dataset are filtered and ranked by using Boruta, LASSO, LightGBM, and mRMR. Finally, the features in 310 datasets are comprehensively evaluated. The intersection of the last three ranked feature lists are taken to obtain essential functional terms that may be highly relevant to the PPI.

This study utilized advanced machine learning methods to investigate relevant functional terms of PPIs. The whole analysis process is illustrated Figure 1. The results generated in each step are then described in detail.

## 3.1 Results of boruta

**FIGURE 2:**
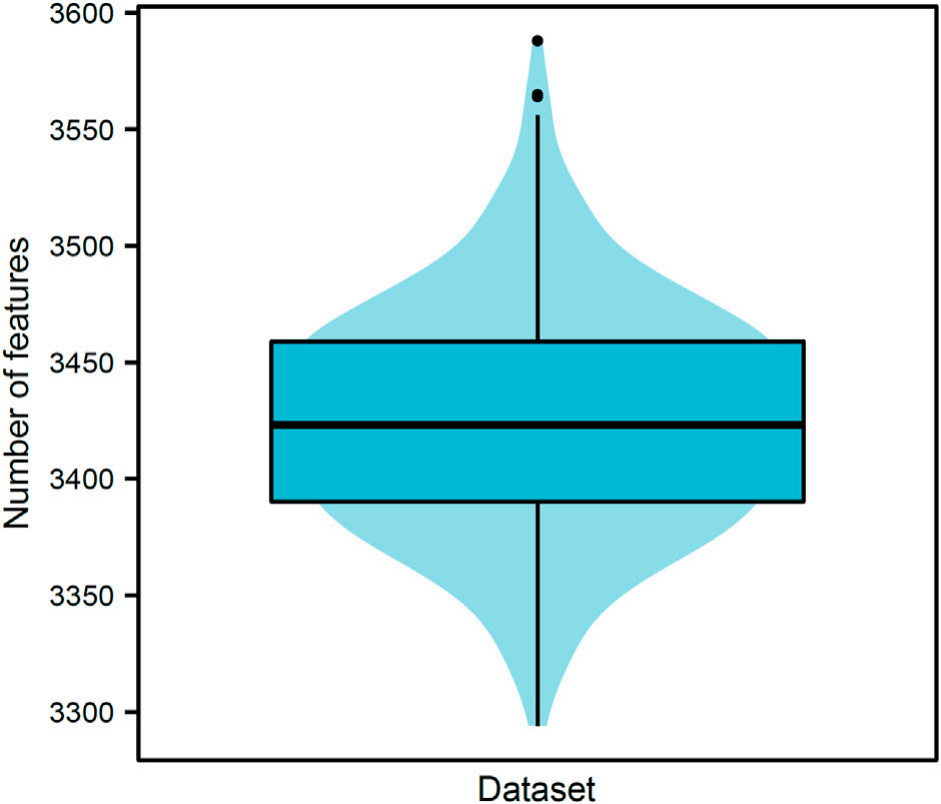
Violin plot of the number of features selected by Boruta on 310 datasets. The numbers of selected features vary from 3200 to 3600, and 3350–3500 features are selected in majority datasets (∼88.06%). This result indicates that the numbers of selected features are not considerably difference despite the different negative samples in different datasets.

Our data included 21,928,753 pairs of 6,623 proteins, where 70,392 were positive samples and rest 21,858,361 were negative samples. Negative samples were divided into 310 parts, thereby constructing 310 datasets. PPIs in each dataset were represented by 17,916 features for GO terms and 279 features for KEGG pathways. For each dataset, all features were analyzed by Boruta. Relevant features were selected. Figure 2 shows the number of selected features from each dataset. The number of selected features ranged from 3200 to 3600 with the median of 3423. The majority of datasets selected 3350–3500 features, suggesting that these numbers did not differ considerably. The detailed features selected from each dataset can be found in Supplementary Table S1. Furthermore, we obtained 5684 different features by combining the selected features derived from 310 datasets, which are provided in Supplementary Table S2. Among these 5684 features, 226 features were about KEGG pathways, whereas 5458 features were about GO terms. These features were used in the subsequent comprehensive assessment.

### 3.2 Results of feature ranking and comprehensive evaluation

Several features were selected by Boruta on each dataset. These features were further analyzed by each feature ranking algorithm, resulting in one feature list. Accordingly, each feature ranking algorithm generated 310 feature lists, which were further integrated into one feature list by comprehensive evaluation method described in Section 2.5. Each of 5684 features was assigned an importance score, which is listed in Supplementary Table S3. The integrated feature list was generated according to above score, which is also provided in Supplementary Table S3.

**FIGURE 3:**
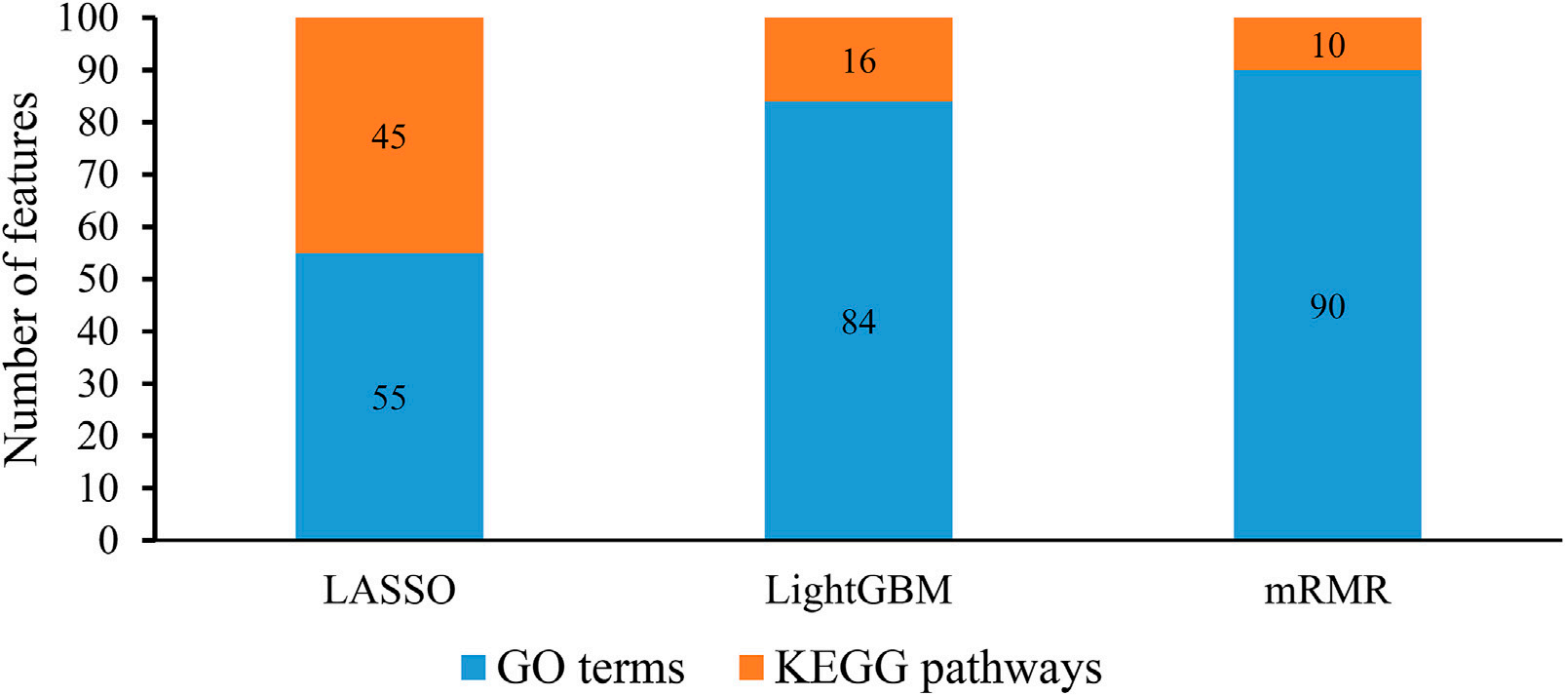
Distribution of top 100 features identified by each feature ranking algorithm on gene ontology (GO) terms and KEGG pathways. The identified features for GO terms are more than those for KEGG pathways.

**FIGURE 4:**
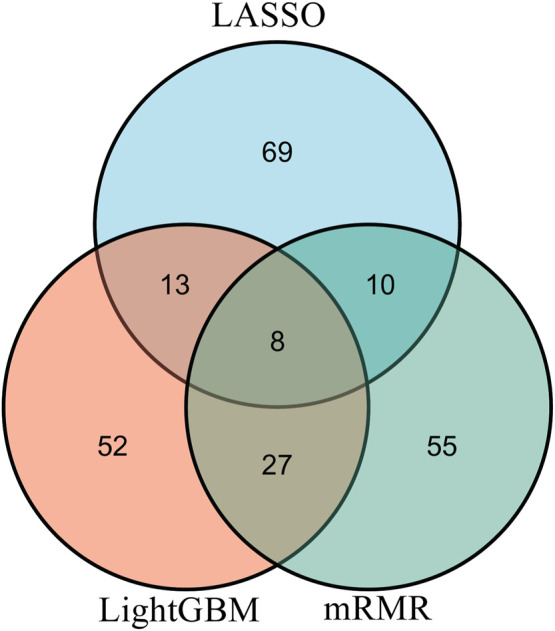
Venn diagram of top 100 features in three integrated feature lists obtained by mRMR, LightGBM, and LASSO methods. The overlapping circles indicate the features that are identified by different ranking algorithms. Eight features are identified and ranked highly by all three feature ranking algorithms.

From each integrated feature list, top 100 features were picked up for further analysis. The distribution of 100 features selected from each integrated list on GO terms and KEGG pathways is provided in Figure 3. It can be observed that features for GO terms were more than those for KEGG pathways regardless of the feature ranking algorithms. However, the quantities were not same. LASSO identified much less features for GO terms than other two methods. By using multiple algorithms, some common functional terms can be discovered and exclusive terms can be mined by a special algorithm. Comprehensive analysis of functional terms identified by three algorithms can make the result more complete. In view of this, the intersection operation was performed on the above three feature subsets selected from the integrated feature lists. A Venn diagram was plotted to show the intersections, as illustrated in Figure 4. The detailed features contained in three, two or one subsets are provided in Supplementary Table S4. Eight features occurred in three subsets, which are listed in Table 1. These features were identified and ranked high by all three feature ranking algorithms, indicating they may provide essential contributions for distinguishing positive and negative samples. At the same time, their corresponding GO terms and KEGG pathways can be used to depict PPIs. Furthermore, 50 features were highly ranked by two algorithms, i.e., they contained in two feature subsets. They may also important for uncovering the essential differences between PPIs and general protein pairs. As for the features contained in one subset, i.e., they were identified by one feature ranking algorithm, they can supplement some exclusive differences between PPIs and general protein pairs, which cannot be uncovered by other algorithms. In Section 4, GO terms and KEGG pathways corresponding to some above features would be discussed.

**TABLE 1:** Eight features with high ranks yielded by all three feature ranking algorithms.

Feature	Description	Group
abs (GO:0031224_1-GO:0031224_2)	Intrinsic component of membrane	Cellular Component
abs (GO:0044425_1-GO:0044425_2)	Obsolete membrane part	Cellular Component
abs (GO:0005615_1-GO:0005615_2)	Extracellular space	Cellular Component
abs (hsa04060_1-hsa04060_2)	Cytokine-cytokine receptor interaction	KEGG pathway
abs (GO:0071944_1-GO:0071944_2)	Cell periphery	Cellular Component
abs (GO:0007186_1-GO:0007186_2)	G protein-coupled receptor Signaling pathway	Biological Process
abs (hsa04514_1-hsa04514_2)	Cell adhesion molecules	KEGG pathway
hsa04060_1 + hsa04060_2	Cytokine-cytokine receptor interaction	KEGG pathway

## 4 Discussion

By using the three feature ranking algorithms of LASSO, LightGBM, and mRMR, we identified some essential biological functional terms that were deemed to be associated with PPIs. We discussed some PPI-associated functional terms identified by using three, two or one algorithms, which are listed in Table 2.

**TABLE 2:** Discussed gene ontology (GO) terms and KEGG pathways.

IDs of GO terms or KEGG pathways	Description	Number of algorithms identified the functional term
GO:0031224	Intrinsic component of membrane	3
hsa04060	Cytokine-cytokine receptor interaction	3
GO:0005515	protein binding	2
hsa04110	Cell cycle	2
GO:0043232	intracellular nonmembrane-bound organelle	1

### 4.1 Key features found by all three feature ranking algorithms

Eight biological functional terms were shown to be associated with the PPIs, which were identified by all three algorithms. The first GO term was intrinsic component of membrane (**GO:0031224**). This term contained multiple functional protein complexes, including anchored component of membrane with PPIs between gp130 and IL-6/IL-6R complex (Narazaki et al., 1993). The linkage of multiple functional PPIs, such as predicted cellular component, to the intrinsic component of membrane validated the efficacy and accuracy of our analysis. Another identified PPI-associated functional term was cytokine–cytokine receptor interaction (**hsa04060**) (Dey et al., 2009), which describes the interaction between membrane-based receptors and soluble cytokines. Considering that cytokines, such as the IL-2, IL-1 and IL-17 family, are small effective soluble proteins, the interactions between cytokines and their respective matched receptors are functional PPIs.

### 4.2 Key features found by any two feature ranking algorithms

Fifty features were identified by exact two algorithms, which involved 48 biological functional terms. The first predicted GO term was a general description of the protein-binding biological process (**GO:0005515**). The next predicted biological function was the cell cycle (**hsa04110**). Recent publications have shown that cell cycle biological processes involve multiple PPIs. The establishment of PPI networks for the cell cycle in *Saccharomyces cerevisiae* early in 2012 confirmed that the cell cycle involves multiple PPIs (Alberghina et al., 2012; Lu et al., 2020). Further studies on human beings and other eukaryotic creatures also validated the role of such identified PPIs in human beings. These PPIs included interactions between TP53 and MDM2 (Lu et al., 2020) and interactions among PDK1, AKT, and the mTOR complex (Pennington et al., 2018). Therefore, the cell cycle is an effective biological process that involves multiple functional PPIs across different eukaryotic species.

### 4.3 Key features found by one of the feature ranking algorithms

Although the remaining 176 features were identified by only one algorithm, some of them may also be important. These features were about 149 functional terms. **GO:0043232** describes intracellular nonmembrane-bound organelle. Few PPIs have been observed to be associated with intracellular nonmembrane-bound organelles. Fewer PPIs may be related to nonmembrane bound organelles than to intracellular membrane-based subcellular structures because biological processes generally involve PPIs, such as cell signaling, immune recognition, and exosome intake, that all depend on biomembrane systems. Therefore, although some pieces of experimental evidence imply that intracellular nonmembrane-bound organelles also involve some PPIs, such as interactions between peptide synthetase and related synthesized proteins (Jaremko et al., 2020).

All in all, as we have discussed above, the biological functional terms predicted by multiple machine learning algorithms have all been confirmed by recent publications with solid experimental support. Therefore, our analyses validated that machine learning algorithms are effective tools for exploring the potential biological functions of PPIs. The application of multiple machine learning algorithms simultaneously may help recognize additional potential PPI-associated functions, thus providing a novel workflow for identifying the biological significance of PPIs.

## 5 Conclusion

In this research, an integrated feature selection method on GO terms and KEGG pathways was established to distinguish significant PPIs. First, Boruta was applied to obtain a set of features that were highly correlated with PPI functions. Three efficient feature ranking algorithms, namely, LASSO, LightGBM, and mRMR, were adopted to rank the filtered features. The intersection of the top-ranked features in three different feature ranking lists was performed to extract most essential GO terms and KEGG pathways. Some essential PPI-associated functional terms, including cytokine–cytokine receptor interaction, intrinsic component of membrane, and protein-binding biological process, were identified. Furthermore, the functional terms mined in our study were analyzed by reviewing the literature.

## Data Availability

Publicly available datasets were analyzed in this study. This data can be found here: https://string-db.org/.
